# Supplementary material for: Intrinsic enzymatic properties modulate the self-propulsion of micromotors
Source: Nat Commun. 2019 Jun 27;10:2826. doi: 10.1038/s41467-019-10726-8 (PMC6597730; doi:10.1038/s41467-019-10726-8)
Supplement: Supplementary file 1 — Supplementary Information [file 41467_2019_10726_MOESM1_ESM.pdf]

Supplementary Information

Intrinsic Enzymatic Properties Modulate the Self-Propulsion of Micromotors

Xavier Arqué, et al.

Supplementary Figures

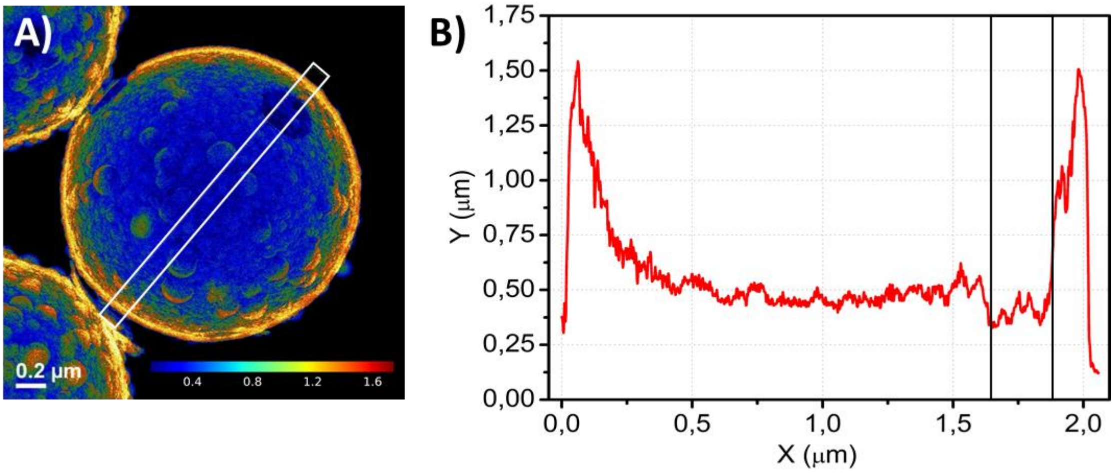

Supplementary Figure 1. Topographic analysis of silica microcapsules. (a) Colored thickness representation of a HSMC taken with TEM Zeiss EM 912 and (b) graphical representation of thickness of a diametrical section of that HSMC. Highlighted area of the hole detected on the thickness representation.

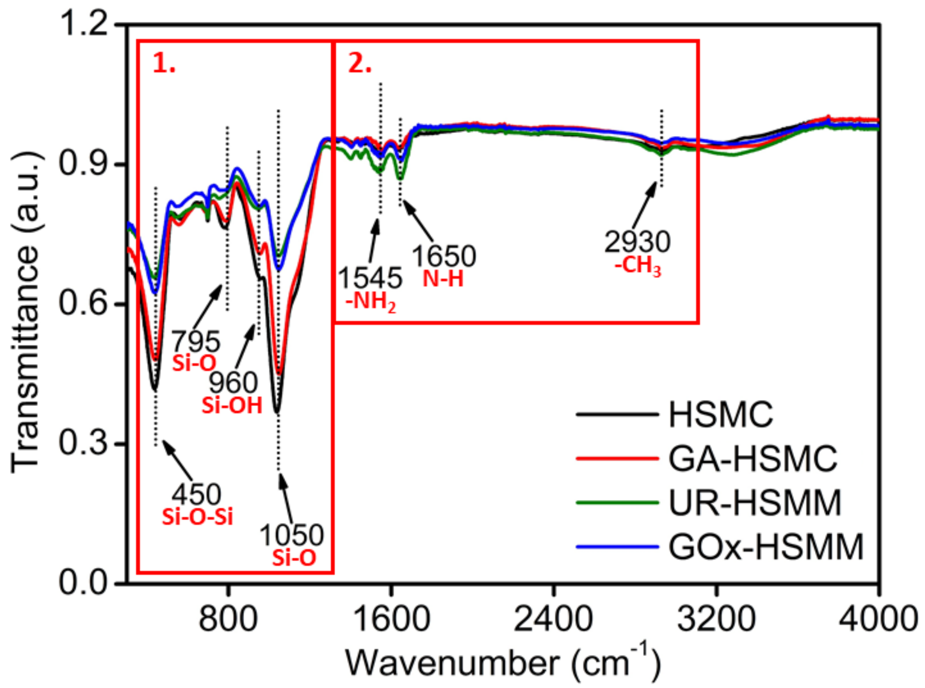

Supplementary Figure 2. Detection of base material composition. FTIR spectra of each silica surface modification step. (1) Signals proving silica as the base material and (2) functional groups of the silica structure. HSMC: Hollow silica microcapsules, GA-HSMC: Hollow silica microcapsules functionalized with glutaraldehyde, UR-HSMM: Hollow silica microcapsules functionalized with urease, GOx-HSMM: Hollow silica microcapsules functionalized with glucose oxidase.

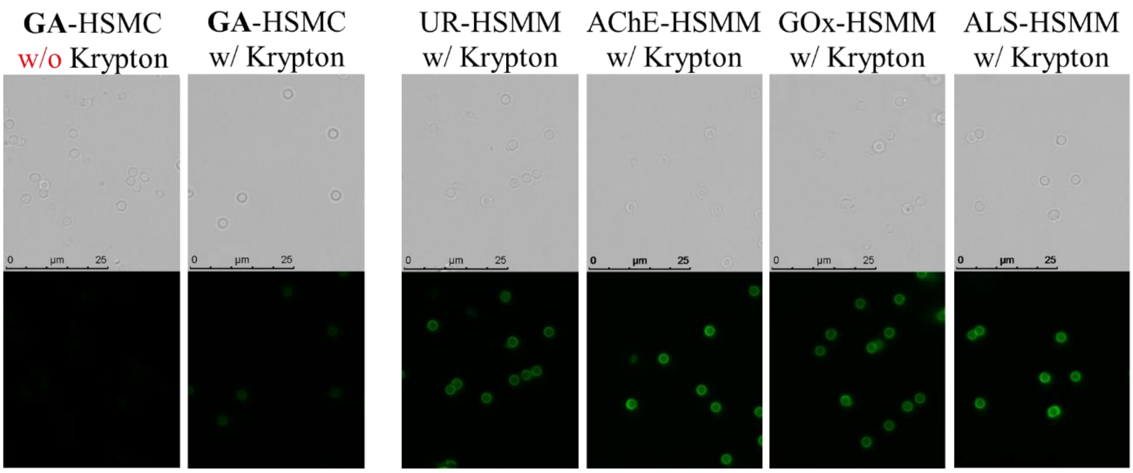

Supplementary Figure 3. Protein labeling on silica microparticles. Bright field and fluorescent (Ex/Em = 520/580 nm) images of HSMC treated with Krypton™ protein staining dye before and after functionalization with enzyme.

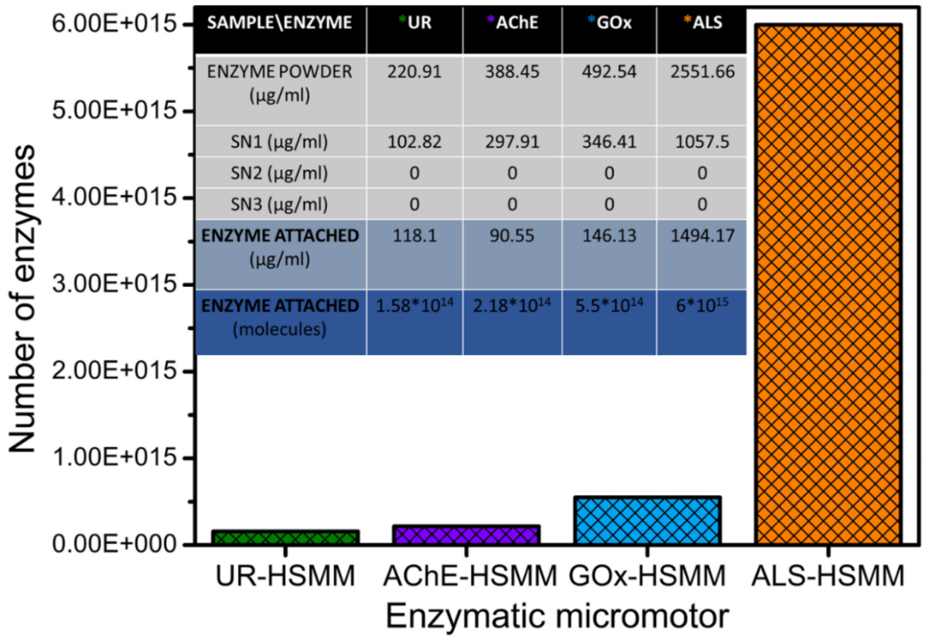

SAMPLE\ENZYME	*UR	*AChE	*GOx	*ALS
ENZYME POWDER (μg/ml)	220.91	388.45	492.54	2551.66
SN1 (μg/ml)	102.82	297.91	346.41	1057.5
SN2 (μg/ml)	0	0	0	0
SN3 (μg/ml)	0	0	0	0
**ENZYME ATTACHED** (μg/ml)	118.1	90.55	146.13	1494.17
**ENZYME ATTACHED** (molecules)	$1.58*10^{14}$	$2.18*10^{14}$	$5.5*10^{14}$	$6*10^{15}$

Supplementary Figure 4. Number of enzymes attached on microparticles. Representation of the number of each of the enzymes attached to hollow silica microcapsules (HSMC) for a functionalization process. Inset: Table of total protein quantification measurements of the enzyme powder used to functionalize for each enzyme and each supernatant discarded after the incubation of the HSMC with the enzymes. Enzyme molecules in the stock powder and attached to the particles are calculated using the molecular weights of the enzymes. Source data are provided as a Source Data file.

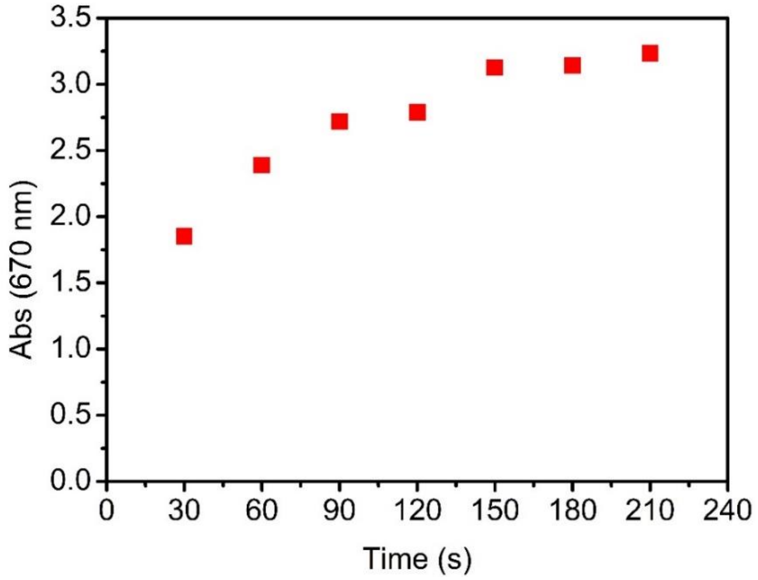

Supplementary Figure 5. Enzymatic activity detection of urease HSMM. Monitoring of ammonia production through increase in absorbance at 670 nm over 3.5 minutes detecting every 30 seconds. Source data are provided as a Source Data file.

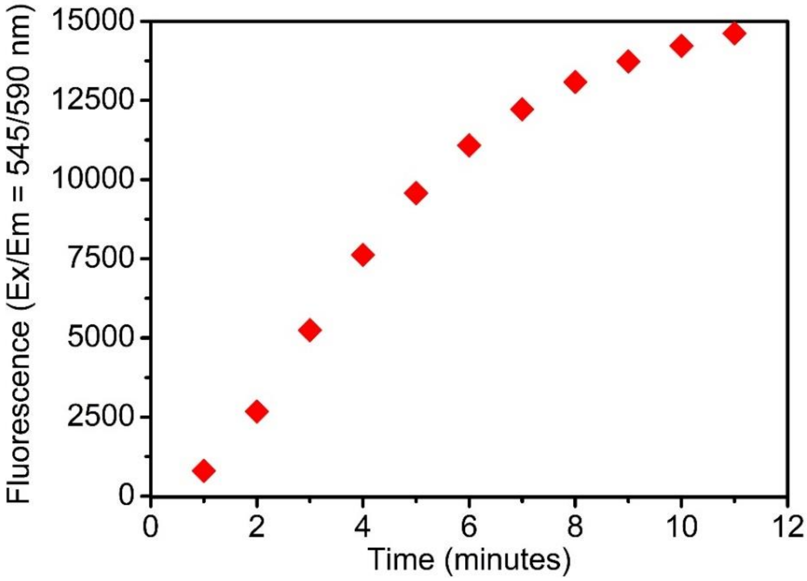

Supplementary Figure 6. Enzymatic activity of acetylcholinesterase HSMM. Monitoring of fluorescence at 585 nm that increases over 11 minutes due to the formation of resorufin, which is, consequence of the acetylcholinesterase enzymatic activity. Source data are provided as a Source Data file.

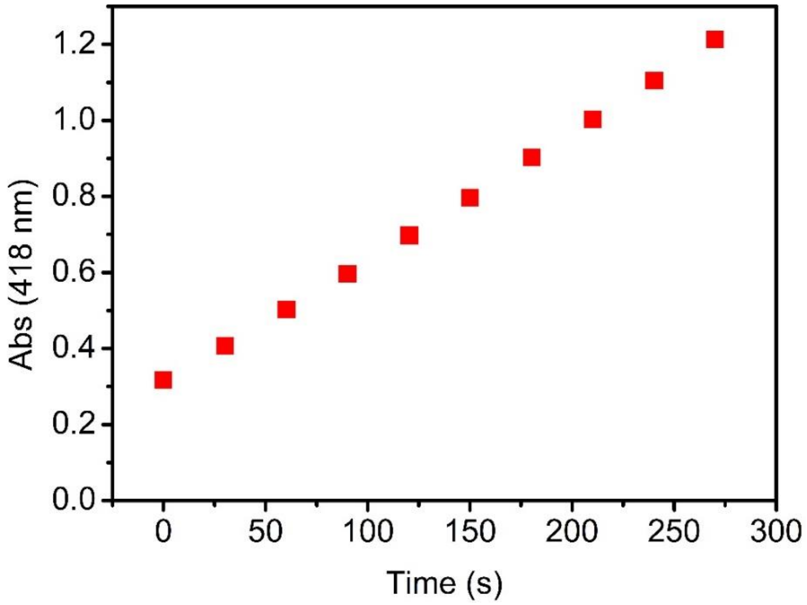

Supplementary Figure 7. Enzymatic activity of glucose oxidase HSMM. Monitoring absorbance at 418 nm increase over 4.5 minutes due to the formation of $ABTS^{2+}$ result of glucose oxidase enzymatic activity. Source data are provided as a Source Data file.

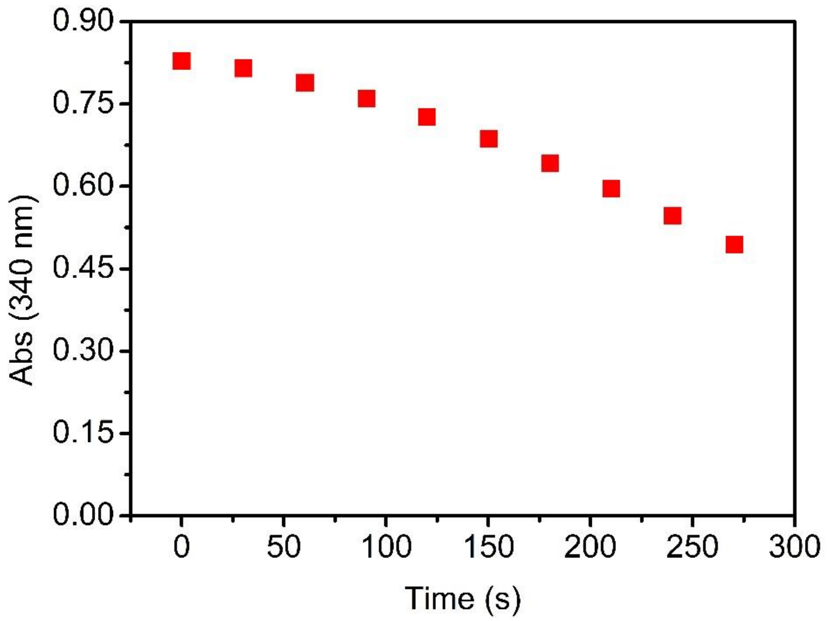

Supplementary Figure 8. Enzymatic activity of aldolase HSMM. Monitoring absorbance at 340 nm decrease over 4.5 minutes due to the oxidation of β-NADH to β-NAD, consequence of the aldolase enzymatic activity. Source data are provided as a Source Data file.

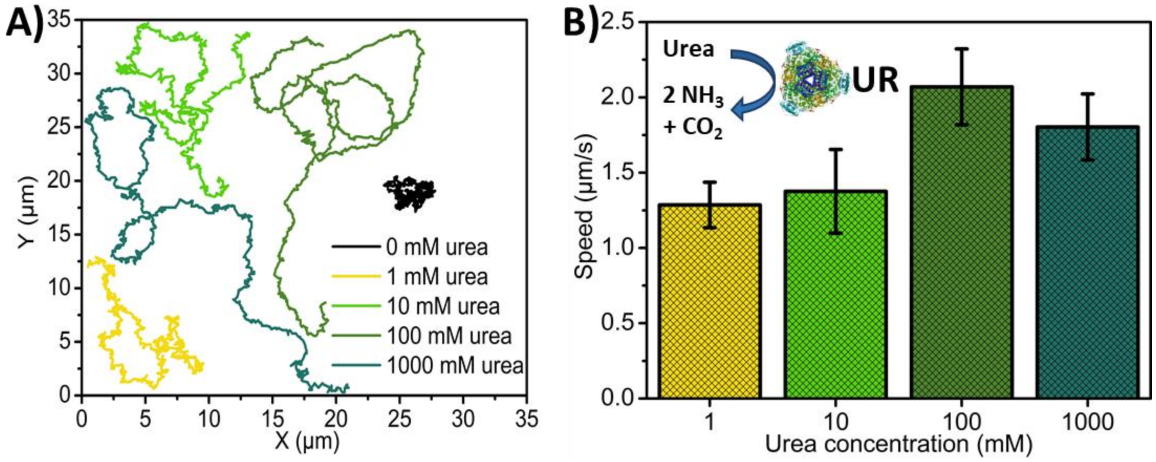

Supplementary Figure 9. Motion dynamics of UR-HSMM. (a) Representative trajectories of UR-HSMM exposed to different urea concentrations. (b) Average velocity of UR-HSMM, extracted from the MSD analysis, for different urea concentrations. Results are shown as the mean ± s.e.m. 20 particles were analysed per condition. Source data are provided as a Source Data file. Enzyme structures are extracted from RCSB PDB (more details in Enzyme Structures Supplementary Notes in Supplementary Information).

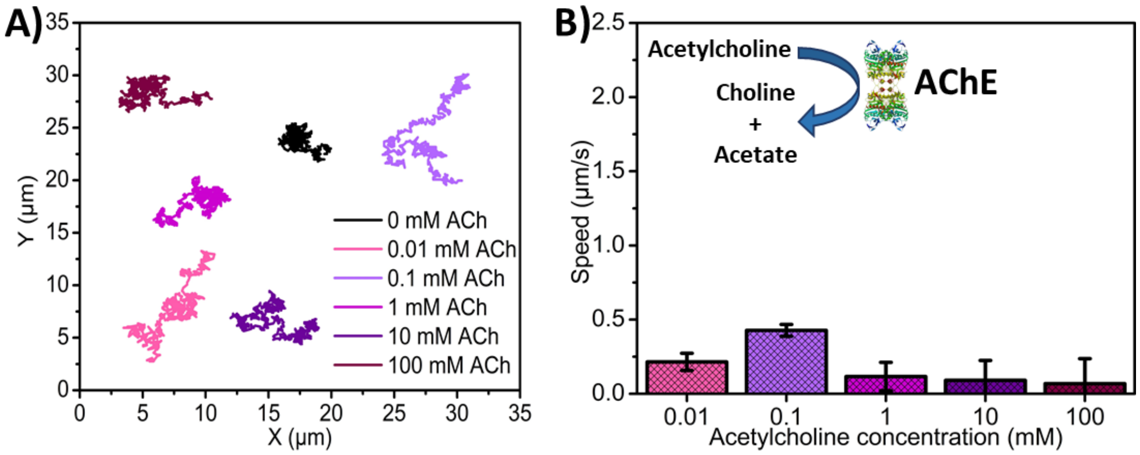

Supplementary Figure 10. Motion dynamics of AChE-HSMM. (a) Representative trajectories of AChE-HSMM exposed to different acetylcholine concentrations. (b) Average velocity of AChE-HSMM, extracted from the MSD analysis, for different acetylcholine concentrations. Results are shown as the mean ± s.e.m. 20 particles were analysed per condition. Source data are provided as a Source Data file. Enzyme structures are extracted from RCSB PDB (more details in Enzyme Structures Supplementary Notes in Supplementary Information).

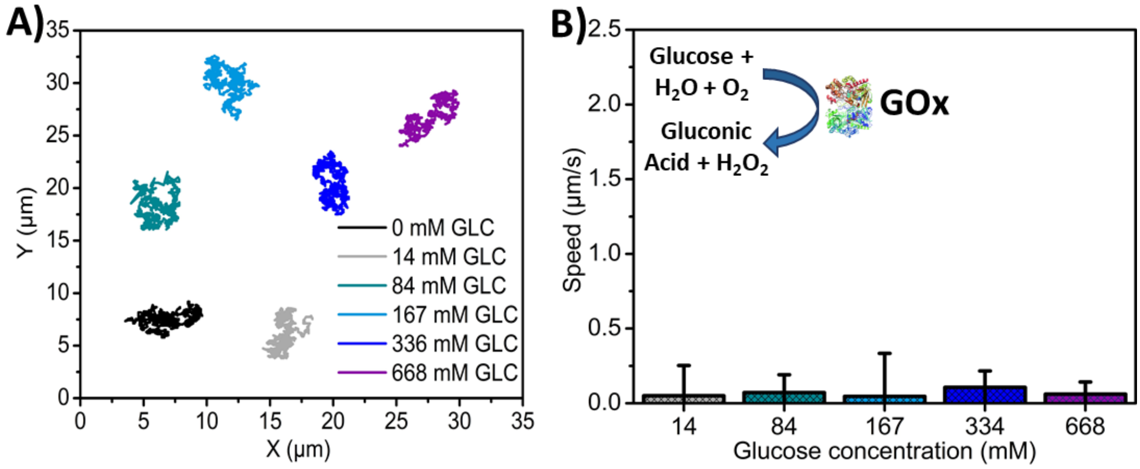

Supplementary Figure 11. Motion dynamics of GOx-HSMM. (a) Representative trajectories of GOx-HSMM exposed to different glucose concentrations. (b) Average velocity of GOx-HSMM, extracted from the MSD analysis, for different glucose concentrations. Results are shown as the mean ± s.e.m. 20 particles were analysed per condition. Source data are provided as a Source Data file. Enzyme structures are extracted from RCSB PDB (more details in Enzyme Structures Supplementary Notes in Supplementary Information).

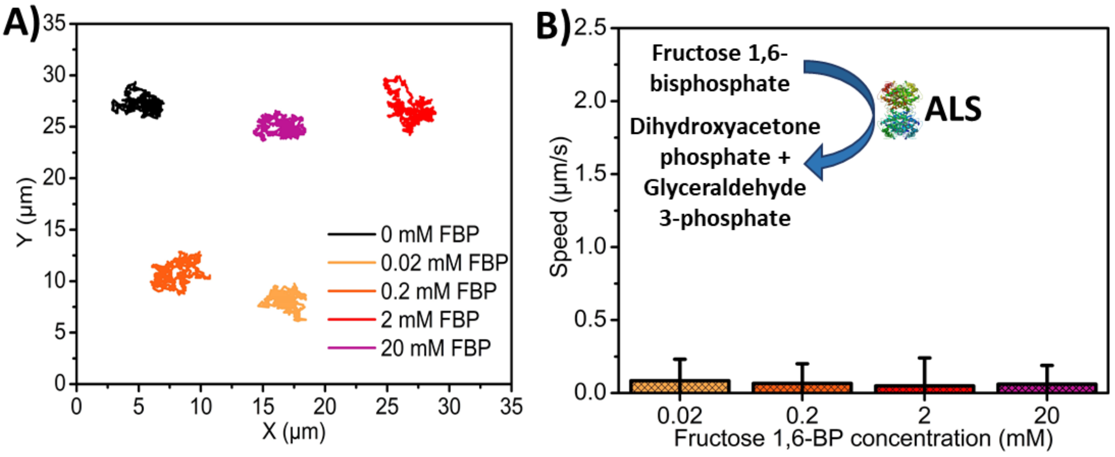

Supplementary Figure 12. Motion dynamics of ALS-HSMM. (a) Representative trajectories of ALS-HSMM exposed to different fructose 1,6-bisphosphate concentrations. (b) Average velocity of ALS-HSMM, extracted from the MSD analysis, for different fructose 1,6-bisphosphate concentrations. Results are shown as the mean ± s.e.m. 20 particles were analysed per condition. Source data are provided as a Source Data file. Enzyme structures are extracted from RCSB PDB (more details in Enzyme Structures Supplementary Notes in Supplementary Information).

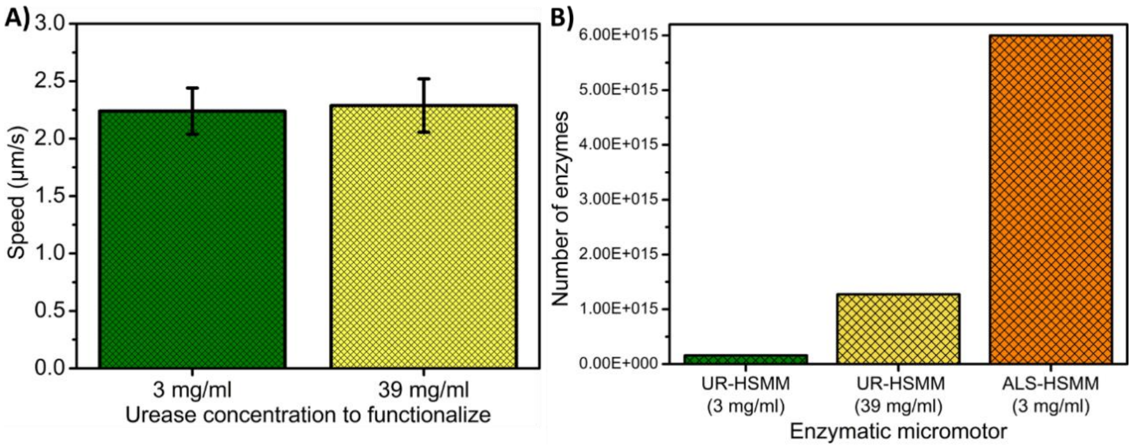

Supplementary Figure 13. Effect of urease quantity on motion dynamics. (a) Speed of UR-HSMM functionalized with (x1) 3 and (x13) 39 mg ml^{-1} exposed to 500 mM urea. (b) Number of enzymes attached to hollow silica microcapsules (HSMC) for UR-HSMM functionalized with 3 (x1) and 39 (x13) mg ml^{-1} and ALS-HSMM functionalized with (x1) 3 mg ml^{-1}. Results are shown as the mean ± s.e.m. 20 particles were analysed per condition. Source data are provided as a Source Data file.

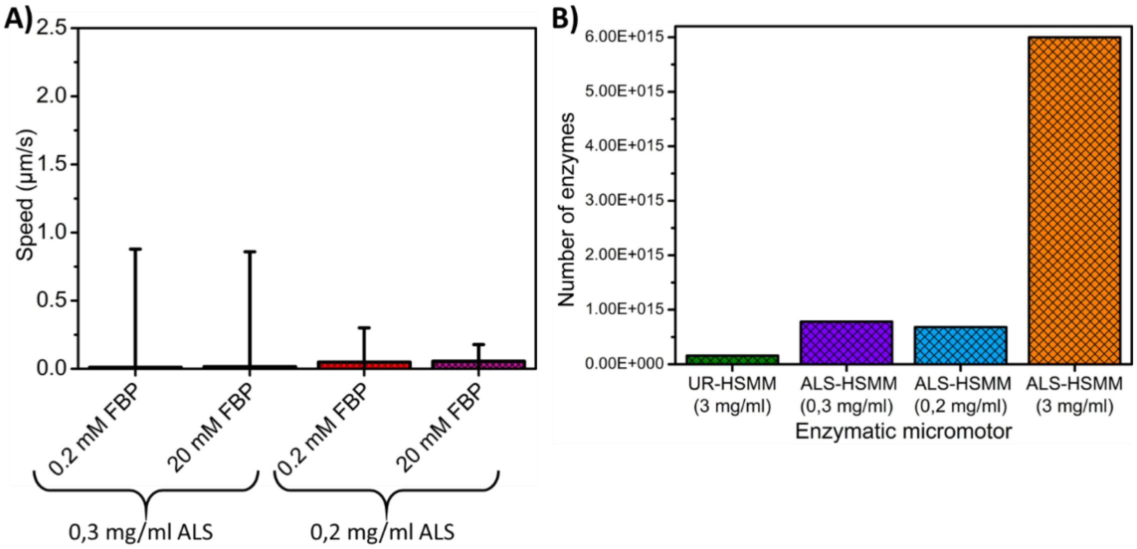

Supplementary Figure 14. Effect of aldolase quantity on motion dynamics. (a) Speed of ALS-HSMM functionalized with (x0.1) 0.3 and (x0.15) 0.2 mg ml^{-1} exposed to 0.2 and 20 mM FBP. (b) Number of enzymes attached to hollow silica microcapsules (HSMC) for ALS-HSMM functionalized with (x1) 3, (x0.1) 0.3 and (x0.15) 0.2 mg ml^{-1} and UR-HSMM functionalized with (x1) 3 mg ml^{-1}. Results are shown as the mean ± s.e.m. 20 particles were analysed per condition. Source data are provided as a Source Data file.

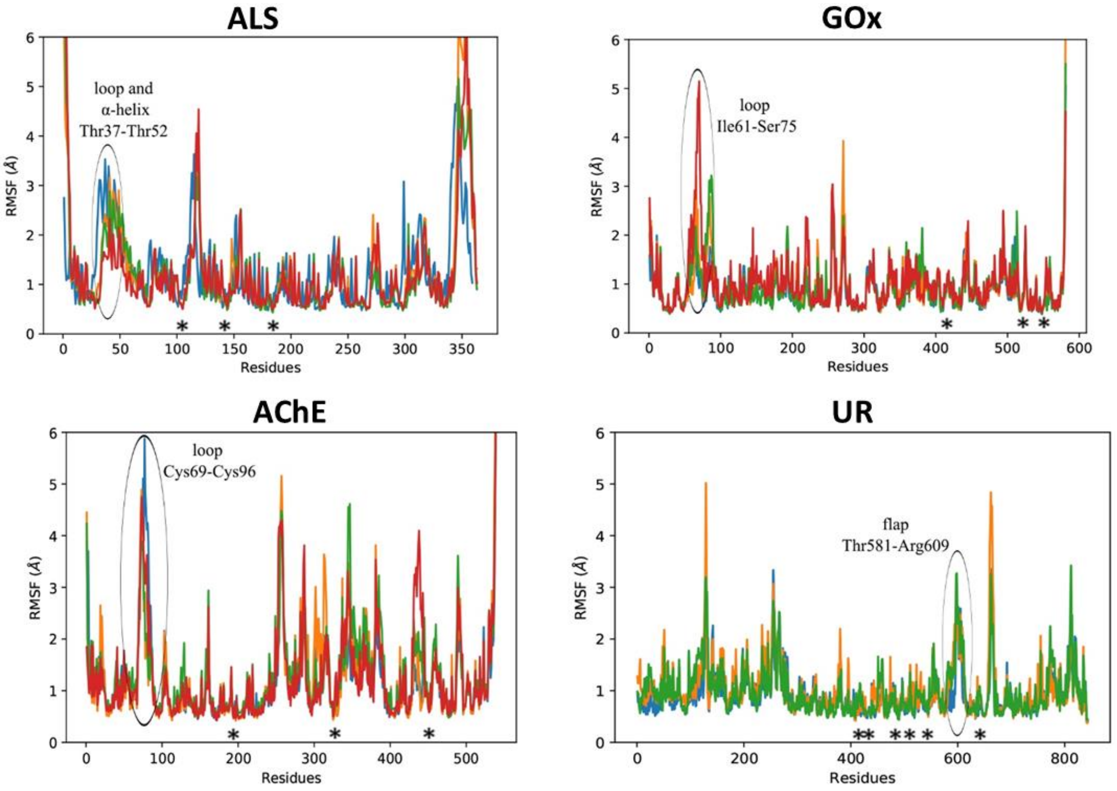

Supplementary Figure 15. Root mean square fluctuation (RMSF) of enzymes. Four replicas are shown for ALS, GOx, and AChE, while urease only one replica with the three monomers is present. Black circles show the flexible regions near the active site suggested for inducing the speediness to the enzymes. Active site residues highlighted with *.

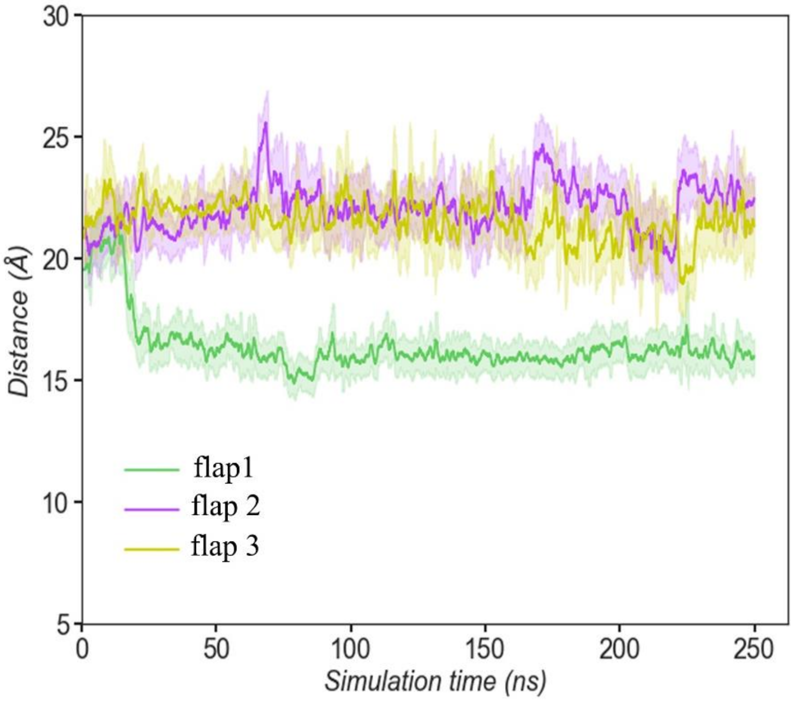

Supplementary Figure 16. Conformations of urease active site flap. Distances of Ala440-Ile599 for measuring the conformation of the flap in the apo state of UR are shown for the three monomers. Open conformations in purple and yellow, and closed conformation in green.

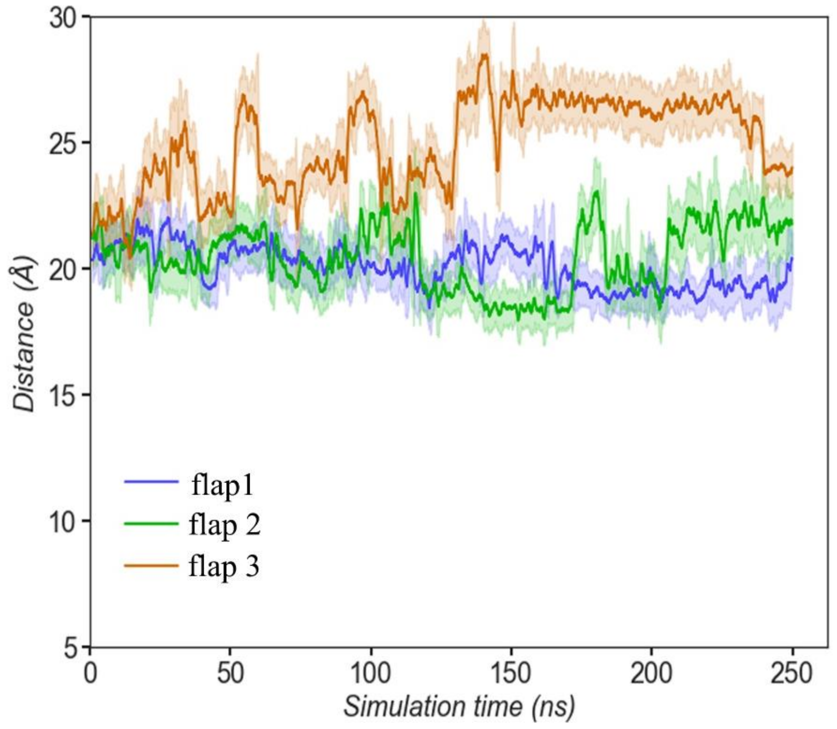

Supplementary Figure 17. Urease active site flap conformation with AHA. Distances of Ala440-Ile599 for measuring the conformation of the flap in presence of AHA in the active site of UR are shown for the three monomers. Open conformations in green and blue, and wide-open conformation in orange.

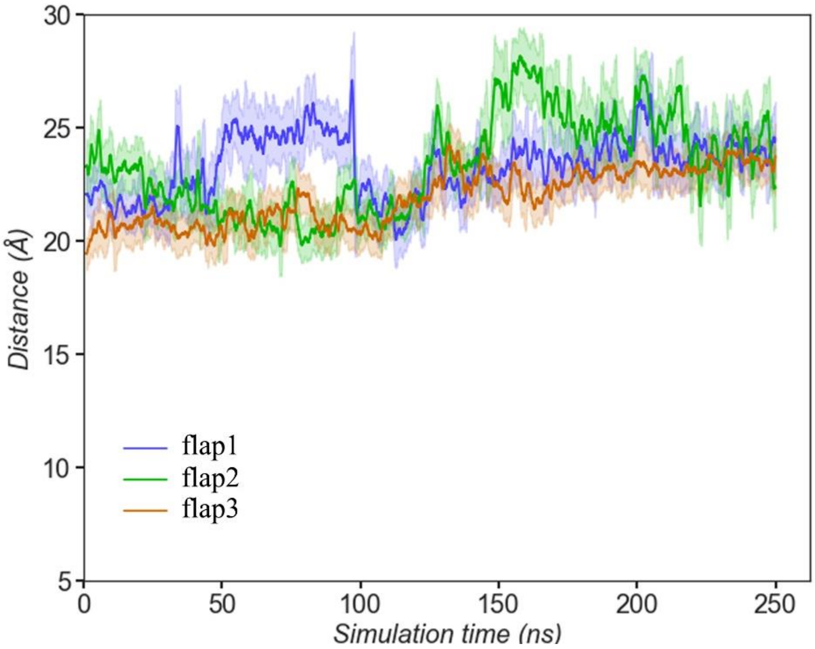

Supplementary Figure 18. Urease active site flap conformation with Cys592-BME. Distances of Ala440-Ile599 for measuring the conformation of the flap in Cys592-BME of UR are shown for the three monomers. Mostly open conformations are explored.

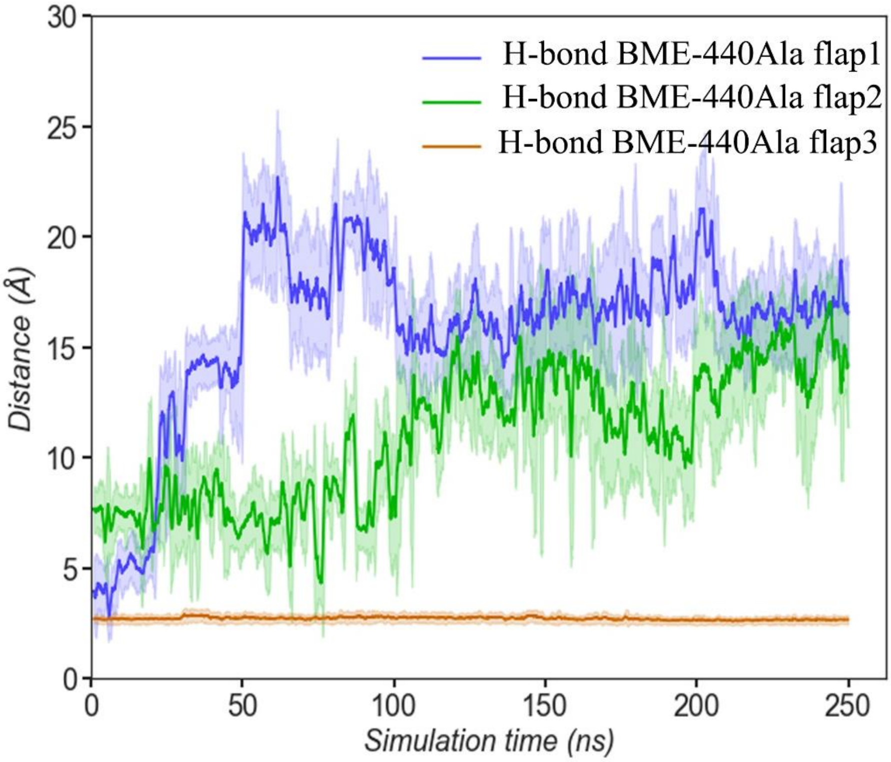

Supplementary Figure 19. H-bond on urease active site flap with Cys592-BME. Distance between (Cys592)-BME and Ala440 of UR shown for the three monomers. One of the monomers shows the H-bond interaction constant during the whole simulation time (see Figure 6 in article).

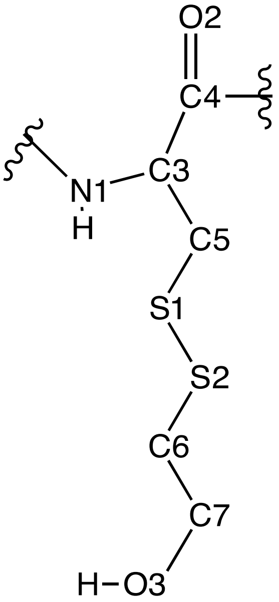

Supplementary Figure 20. Structural formula of Cys592-BME modified residue. Atom names of Cys592-BME modified residue in Supplementary Table 1.

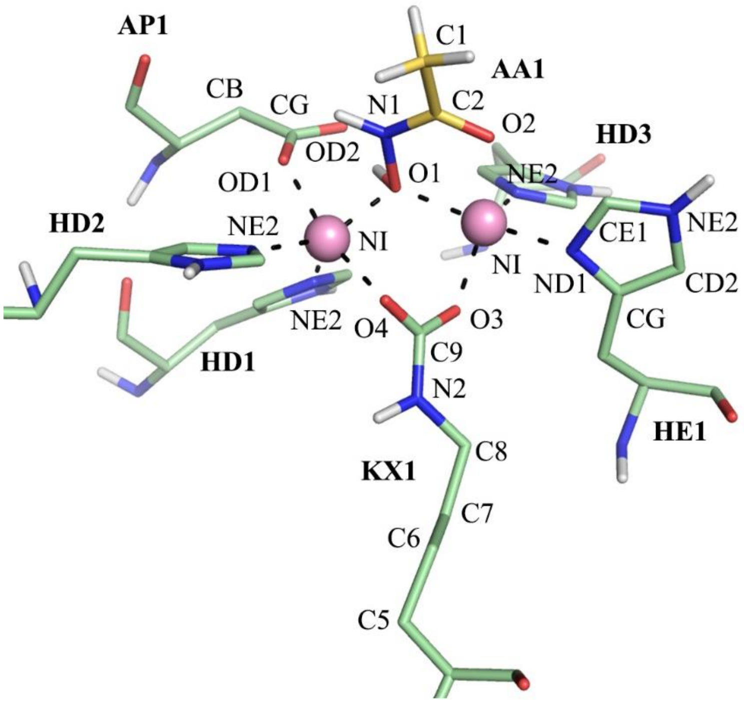

Supplementary Figure 21. 3D structural representation of urease nickel active site. Atom names of the urease nickel active site used to obtain the bonded parameters of urease in Supplementary Data 1, Page 1: Atomic Charges.

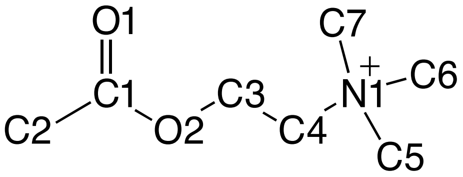

Supplementary Figure 22. Structural formula of acetylcholine. Atom names of acetylcholine in Supplementary Table 2.

Supplementary Tables

Supplementary Table 1. Cys592-BME atom types and charges for performing the MD simulations in Supplementary Figure 20.

Atom name	Atom type	Charge	Atom name	Atom type	Charge
N1	n	-0.738200	C5	c3	-0.238863
H3	hn	0.405759	H5	h1	0.166628
C3	c3	0.059617	H6	h1	0.166628
C4	c	0.422487	C6	c3	0.128044
O2	o	-0.516729	H7	h1	-0.002164
N2	n	-0.293024	H8	h1	-0.002164
H2	hn	0.289496	C7	c3	0.195073
C8	c3	-0.092738	H9	h1	0.037766
H12	h1	0.081178	H10	h1	0.037766
H13	h1	0.081178	O3	oh	-0.680133
H14	h1	0.081178	H11	ho	0.426561
H4	h1	0.154565	S2	ss	-0.139983
S1	ss	-0.156477			

Supplementary Table 2. Acetylcholine atom types and charges for performing the MD simulations in Supplementary Figure 22.

Atom name	Atom type	Charge	Atom name	Atom type	Charge
O1	o	-0.560759	N1	n4	0.190678
C1	c	0.824598	C6	c3	-0.394927
C2	c3	-0.581564	H11	hx	0.197045
H1	hc	0.175806	H12	hx	0.197045
H2	hc	0.175806	H13	hx	0.197045
H3	hc	0.175806	C7	c3	-0.394927
O2	os	-0.480099	H14	hx	0.197045
C3	c3	0.438196	H15	hx	0.197045
H4	h1	0.000454	H16	hx	0.197045
H5	h1	0.000454	C5	c3	-0.394927
C4	c3	-0.310102	H8	hx	0.197045
H6	hx	0.181049	H9	hx	0.197045
H7	hx	0.181049	H10	hx	0.197045

Supplementary Notes

Supplementary Note 1. Materials and Instruments. 2 µm micro particles based on polystyrene (Sigma-Aldrich cat. no. 78452), ethanol 99% (Panreac Applichem cat. no. 131086-1214), ammonium hydroxide solution (Sigma-Aldrich cat. no. 221228), 3-aminopropyltriethoxysilane (APTES) 99% (Sigma-Aldrich cat. no. 440140), tetraethylorthosilicate (TEOS) ≥99% (Sigma-Aldrich cat. no. 86578), dimethylformamide (DMF) ≥99.8% (Acros Organics cat. no. 423640010), 1X Phosphate-buffered saline (PBS) (pH = 7.4) (Thermo Fischer Scientific cat. no. 70011-036), glutaraldehyde (GA) (25 wt %) (Sigma-Aldrich cat. no. G6257), urease from *Canavalia ensiformis* (Jack bean) (Sigma-Aldrich cat. no. U4002), glucose oxidase from *Aspergillus niger* (Sigma-Aldrich cat. no. G2133), aldolase from *Oryctolagus cuniculus* (Rabbit) (Sigma-Aldrich cat. no. A2714), acetylcholinesterase from *Electrophorus electricus* (Electric eel) (Sigma-Aldrich cat. no. C2888), acetylcholine (ACh) (Sigma-Aldrich cat. no. A6625), Krypton™ protein staining (Thermo Scientific cat. no. 46628), Pierce™ BCA Protein Assay Kit (Thermo Fisher cat. no. 23227), Urease Activity Assay Kit (Sigma-Aldrich cat. no. MAK120), urea (Sigma-Aldrich cat. no. U5128), acetohydroxamic acid (AHA) (Sigma-Aldrich cat. no. 159034), Amplex® Red Acetylcholine/Acetylcholinesterase Assay Kit (Thermo Fisher Scientific A12217), of glucose (GLC) (Sigma-Aldrich cat. no. G5767), 2,2'-azino-bis(3-ethylbenzothiazoline-6-sulphonic acid) (ABTS) (Sigma-Aldrich cat. no. A1888), horseradish peroxidase (HRP) (Sigma-Aldrich cat. no. P8250), fructose 1,6-bisphosphate (FBP) (Sigma-Aldrich cat. no. F6803), triosephosphate isomerase (TPI)/ α-glycerophosphate dehydrogenase (α-GDH) (Sigma-Aldrich cat. no. G1881), β-nicotinamide adenine dinucleotide (β-NAD) (Sigma-Aldrich cat. no. N8129), β-mercaptoethanol (BME) (Sigma-Aldrich cat. no. M6250). The optical videos and the fluorescent images of the enzymatic micromotors motion were recorded using the camera (Hamamatsu Digital Camera C11440-22C) of an inverted optical microscope (Leica DMi8). SEM images were captured by a FEI NOVA NanoSEM 230. The absorbance measurements were done with the Benchmark Plus Micro Platereader from Bio-Rad. The fluorescence measurements were done with the BioTek FLX800TBIE. TEM images were captured by a Zeiss EM 912. The Fourier-transformed infrared spectroscopy measurements were performed with the Perkin Elmer Frontier.

Supplementary Note 2. Briggs-Haldane kinetics. The separation between association-dissociation processes and catalysis can be better understood by the Briggs-Haldane schematic, a derivation of the Michaelis-Menten model,

$$\mathrm{E}+\mathrm{S} \underset{k_{\mathrm{S\cdot off}}}{\overset{k_{\mathrm{S\cdot on}}}{\rightleftarrows}} \mathrm{ES} \overset{k_{\mathrm{cat}}}{\longrightarrow} \mathrm{E}+\mathrm{P}, \tag{1}$$

being E the enzyme, S the substrate, ES the enzyme-substrate complex, $k_{S\cdot on}$ and $k_{S\cdot off}$ the association and dissociation rate constants of the substrate to the active site, respectively, k_{cat} the turnover number and *P* the product. When there is also a reversible competitive inhibitor present the enzyme can interact with it, but without catalysis taking place:

$$\mathrm{E}+\mathrm{I} \underset{k_{\mathrm{I\cdot off}}}{\overset{k_{\mathrm{I\cdot on}}}{\rightleftarrows}} \mathrm{EI} \tag{2}$$

where I is the inhibitor, EI the inhibitor-enzyme complex, $k_{I\cdot on/off}$ the association and dissociation rate constants of the inhibitor to the active site.

Supplementary Note 3. Enzyme Structures. The enzymatic structures used in Figure 1, 2, 3, 6 and 7 are extracted from RSCB PDB: urease,[1] acetylcholinesterase,[2] glucose oxidase[3] and aldolase.[4]

Supplementary Note 4. Fourier-transformed infrared spectroscopy (FTIR). The silica surface modification process was monitored by Fourier-transform infrared spectroscopy (FTIR) measurements (Supplementary Figure 2). The peak at 450 cm^{-1} was assigned to the vibration of Si-O-Si bonds, and the presence of peaks at 795 cm^{-1} and 1050 cm^{-1}, assigned to the symmetric and asymmetric vibrations of the Si-O bond, respectively, suggested that the micromotors were based on silicon dioxide. Non-condensed hydroxyl groups (-OH) from Si-OH bond were present as indicated by the peak at 960 cm^{-1}. Additionally, the peak at 2930 cm^{-1}, assigned to the vibration of $-CH_3$ groups corresponded to the silica precursors, TEOS and APTES. Furthermore, the vibration peaks at 1545 cm^{-1} and 1650 cm^{-1} of N-H bonds and $-NH_2$ groups that appear on HSMC and GA-HSMC proved the presence APTES silica precursor. Both methyl and primary amino groups were also due to enzyme conjugation.[5]

Supplementary Note 5. Fluorescent Krypton™ protein staining. The fluorescent (Ex/Em = 520/580 nm) images in Supplementary Figure 3 indicate the presence of proteins after functionalization of the silica surface with urease, acetylcholinesterase, glucose oxidase and aldolase. Although the GA-HSMC treated with protein staining also showed fluorescence due to a nonspecific reaction of the staining, the intensity was much lower. On the other hand, the fluorescence that the glutaraldehyde molecule emits inherently was not responsible for the signal, since it was not visible for the untreated GA-HSMC.

Supplementary Note 6. Total protein quantification before and after functionalization. The unattached enzyme was detected and by analyzing the purity of the enzyme powder mass used to functionalize (3000 µg/ml for urease, glucose oxidase and aldolase, and 1000 µg/ml for acetylcholinesterase), the enzyme attached per functionalization process was calculated. The protein quantified in the first supernatant (SN1) extracted after functionalization was subtracted to the protein quantified in the enzyme powder, since all the unattached enzyme was eliminated in this first supernatant and the following (SN2 and SN3) did not show presence of protein. The remarkable presence of enzyme after the functionalization in Figure 3a and Supplementary Figure 4 suggested that the enzyme is in excess when initially mixed with the HSMC, since much shorter mixing times than an overnight process has been reported for this reaction.[6] The molecular weights were used to calculate the number of enzyme molecules that were attached.

Supplementary Note 7. Activity detection of urease hollow silica micromotors. The activity in ultrapure water of urease attached to HSMC was evaluated using the Urease Activity Assay Kit (Sigma-Aldrich cat. no. MAK120) based on the Berthelot method.[7] The process followed is detailed in the *Technical Bulletin of the Urease Activity Assay Kit*. It works through the ammonia generated (Supplementary Equation 3) by the catalysis of urea (Sigma-Aldrich cat. no. U5128) by urease and monitoring the absorbance at 670 nm.[7] The enzymatic reaction is the following:

$$\text{urea} \xrightarrow{\text{urease}} 2\ \text{NH}_2 + \text{CO}_2 \qquad (3)$$

The process followed is detailed in the *Technical Bulletin of the Urease Activity Assay Kit*. The enzymatic activity was investigated over time, by incubating the UR-HSMM with urea for 3.5 min and analyzing the signal every 30 seconds through plate reader UV-

spectrophotometry, using the urea solution provided in the kit dissolved in ultrapure water. The increase in absorbance (due to ammonia production) in function of time is depicted in Supplementary Figure 7.

The same protocol was followed to study the urease enzymatic activity exposed to different concentrations of acetohydroxamic acid (0 mM, 0.06 mM, 0.7 mM, 6 mM and 50 mM) (Sigma-Aldrich cat. no. 159034) and different concentrations of β-mercaptoethanol (0 mM, 0.5 mM, 5 mM and 50 mM) (Sigma-Aldrich cat. no. M6250), respectively, with urea (Sigma-Aldrich cat. no. U5128) present in excess (500 mM) all dissolved in ultrapure water, for 3 minutes of reaction.

Supplementary Note 8. Activity detection of acetylcholinesterase hollow silica micromotors. The activity in ultrapure water of acetylcholinesterase attached to HSMC was investigated by using the Amplex® Red Acetylcho-line/Acetylcholinesterase Assay Kit (Thermo Fisher Scientific A12217) which detects the choline production from the hydrolysis of acetylcholine catalyzed by the AChE-HSMM (Supplementary Equation 4).

$$\text{acetylcholine} \xrightarrow{\text{acetylcholinesterase}} \text{acetic acid} + \text{choline} \tag{4}$$

Choline is oxidized by choline oxidase to betaine and H_2O_2 (Supplementary Equation 5), the latter of which, in presence of horseradish peroxidase, reacts with 10-acetyl-3,7-dihydroxyphenoxazine (Amplex Red reagent) to generate the highly fluorescent product resorufin (Supplementary Equation 6).

$$\text{choline} + 2\text{O}_2 + \text{H}_2\text{O} \xrightarrow{\text{choline oxidase}} \text{betaine} + 2\text{H}_2\text{O}_2 \tag{5}$$

$$\text{Amplex Red Reagent} + \text{H}_2\text{O}_2 \xrightarrow{\text{horseradish peroxidase}} \text{resorufin} + \text{H}_2\text{O} \tag{6}$$

This molecule has absorption and fluorescence emission maximum at approximately 571 nm and 585 nm, respectively. The process followed is detailed in the manual from the Amplex® Red Acetylcholine/Acetylcholinesterase Assay Kit The increase in fluorescence was tracked every minute for a total of 11 minutes as shown in Supplementary Figure 8.

Supplementary Note 9. Activity detection of glucose oxidase hollow silica micromotors. The activity in ultrapure water of glucose oxidase attached to HSMC was checked by the initial oxidation of glucose (GLC) (Sigma-Aldrich cat. no. G5767) by glucose oxidase resulting in gluconic acid and hydrogen peroxide. Then, this hydrogen

peroxide is used to oxidize the 2,2'-azino-bis(3-ethylbenzothiazoline-6-sulphonic acid) (ABTS) (Sigma-Aldrich cat. no. A1888) molecule by horseradish peroxidase (HRP) (Sigma-Aldrich cat. no. P8250) to obtain $ABTS^{2+}$ (turquoise dye) which absorbance at 418 nm was monitored over time. This chained reaction is depicted in Supplementary Equations 7 and 8.

$$\mathrm{D-glucose} + \mathrm{O_2} + \mathrm{H_2O} \xrightarrow{\text{glucose oxidase}} \mathrm{D-gluconic\ acid} + \mathrm{H_2O_2} \qquad (7)$$

$$\mathrm{ABTS} + \mathrm{H_2O_2} \xrightarrow{\text{horseradish peroxidase}} \mathrm{ABTS^{2+}} + \mathrm{H_2O} \qquad (8)$$

To detect glucose oxidase activity, 500 µl of 1 M GLC (180 mg ml^{-1}), 500 µl of ABTS 1.943 mM (1 mg ml^{-1}) and 100 µl of HRP solution (2 mg ml^{-1}) were placed in a cuvette. All solutions were prepared in ultrapure water. Then, 20 µl of GOx-HSMM solution was added and mixed. The absorbance at 418 nm was monitored through UV-spectrophotometry for 4.5 minutes every 30 seconds as it is shown in Supplementary Figure 9.

Supplementary Note 10. Activity detection of aldolase hollow silica micromotors. The activity in ultrapure water of aldolase attached to HSMC was confirmed by means of a chained reaction starting with the conversion of fructose 1,6-bisphosphate (FBP) (Sigma-Aldrich cat. no. F6803) and water to dihydroxyacetone phosphate (DHAP) and glyceraldehyde 3-phosphate (G3-P) by the catalytic activity of aldolase from the ALS-HSMM. The G3-P obtained is converted by triosephosphate isomerase (TPI) (Sigma-Aldrich cat. no. G1881) to DHAP as well. These reactions are represented in the Supplementary Equations 9 and 10:

$$\mathrm{FBP} + \mathrm{O_2} + \mathrm{H_2O} \xrightarrow{\text{aldolase}} \mathrm{G3-P} + \mathrm{DHAP} \qquad (9)$$

$$\mathrm{G3-P} \xrightarrow{\text{TPI}} \mathrm{DHAP} \qquad (10)$$

Then, the DHAP produced by these two reactions is catalyzed by α-glycerophosphate dehydrogenase (α-GDH) (Sigma-Aldrich cat. no. G1881) with the reduced form of β-nicotinamide adenine dinucleotide (β-NADH) to obtain the oxidized β-nicotinamide adenine dinucleotide (β-NAD) (Sigma-Aldrich cat. no. N8129) and α-glycerophosphate. This reaction is depicted in the Supplementary Equation 11 and the decrease of β-NADH was monitored by detecting its absorbance at 340 nm:

$$2\,\mathrm{DHAP} + 2\,\beta-\mathrm{NADH} \xrightarrow{\alpha-\mathrm{GDH}} 2\,\alpha-\mathrm{glycerophosphate} + 2\,\beta-\mathrm{NAD} \qquad (11)$$

To detect aldolase activity, 800 μl of ultrapure water was mixed in a cuvette with 33.3 μl of 60 mM FBP, 33.3 μl of 4 mM β-NADH and 33.3 μl of α-GDH/TPI solution (from a stock containing 50 units/ml). All solutions were prepared in ultrapure water. Then, 100 μl of ALS-HSMM solution were added and mixed. The absorbance at 340 nm was monitored through UV-spectrophotometry for 4.5 minutes every 30 seconds as it is shown in the Supplementary Figure 10.

Supplementary References

1. Balasubramanian, A. & Ponnuraj, K. Crystal structure of the first plant urease from jack bean: 83 years of journey from its first crystal to molecular structure. *J. Mol. Biol.* **400,** 274–283 (2010).
2. Bourne, Y., Grassi, J., Bougis, P. E. & Marchot, P. Conformational flexibility of the acetylcholinesterase tetramer suggested by x-ray crystallography. *J. Biol. Chem.* **274,** 30370–30376 (1999).
3. Ghoshdastider, U. *et al.* Molecular effects of encapsulation of glucose oxidase dimer by graphene. *RSC Adv.* **5,** 13570–13578 (2015).
4. Herzik, M. A., Wu, M. & Lander, G. C. Achieving better-than-3-Å resolution by single-particle cryo-EM at 200 keV. *Nat. Methods* **14,** 1075–1078 (2017).
5. Pretsch, E., Bühlmann, P., Affolter, C., Herrera, A. & Martínez, R. *Structure Determination of Organic Compounds*. (MASSON, S.A, 2000).
6. López-Gallego, F. *et al.* Enzyme stabilization by glutaraldehyde crosslinking of adsorbed proteins on aminated supports. *J. Biotechnol.* **119,** 70–75 (2005).
7. Patton, C. J. & Crouch, S. R. Spectrophotometric and kinetics investigation of the Berthelot reaction for the determination of ammonia. *Anal. Chem.* **49,** 464–469 (1977).
