# Supplementary material for: Intrinsic enzymatic properties modulate the self-propulsion of micromotors
Source: Nat Commun. 2019 Jun 27;10:2826. doi: 10.1038/s41467-019-10726-8 (PMC6597730; doi:10.1038/s41467-019-10726-8)
Supplement: Supplementary file 3 — Description of Additional Supplementary Files [file 41467_2019_10726_MOESM3_ESM.pdf]

Description of Additional Supplementary Files

File name: Supplementary Data 1

Description: Parameters and charges of urease obtained at B3LYP/6-31G* level of theory. Atomic partial charges for all residues of urease highlighted in Supplementary Figure 21; atom types, masses, and Lennard-Jones parameters for urease metal center; bond parameters for urease; dihedral angle parameters for urease; and improper dihedral parameters for urease.

File name: Supplementary Movie 1

Description: Motion of UR-HSMM exposed to different concentrations of urea.

File name: Supplementary Movie 2

Description: Motion of AChE-HSMM exposed to different concentrations of acetylcholine (ACh).

File name: Supplementary Movie 3

Description: Motion of GOx-HSMM exposed to different concentrations of glucose (GLC).

File name: Supplementary Movie 4

Description: Motion of ALS-HSMM exposed to different concentrations of fructose 1,6-bisphosphate (FBP).

File name: Supplementary Movie 5

Description: Conformational dynamics and tunnel to access active site of AChE.

File name: Supplementary Movie 6

Description: Conformational dynamics and tunnel to access active site of UR.

File name: Supplementary Movie 7

Description: Motion of UR-HSMM exposed to different concentrations of AHA when exposed to 500 mM urea.

File name: Supplementary Movie 8

Description: Motion of UR-HSMM exposed to different concentrations of AHA.

File name: Supplementary Movie 9

Description: Motion of UR-HSMM exposed to different concentrations of BME when exposed to 500 mM urea.
